# Association between adherence to the Mediterranean Diet and the Eatwell Guide and changes in weight and waist circumference in post-menopausal women in the UK Women’s Cohort Study

**DOI:** 10.1177/20533691231156643

**Published:** 2023-02-04

**Authors:** Nicola Best, Orla Flannery

**Affiliations:** Faculty of Health and Education, 5289 Manchester Metropolitan University, Manchester, UK

**Keywords:** Postmenopause, waist circumference, abdominal obesity, Eatwell Guide, Mediterranean Diet

## Abstract

**Objective:**

This study investigated the associations between adherence to the Mediterranean Diet and the Eatwell Guide (EWG) and changes in weight and waist circumference in post-menopausal women.

**Study Design:**

Post-hoc analysis of post-menopausal women from the UK Women’s Cohort Study.

**Main outcome measures:**

Changes in weight, waist circumference and the risk of abdominal and general obesity.

**Results:**

4162 post-menopausal women were selected. Higher adherence to both the EWG and the Mediterranean Diet was associated with smaller increases in waist circumference over 4 years (EWG: β −0.47, CI −0.75, −0.20 per 1 tertile increase in score), (Mediterranean Diet: β −0.29, CI −0.58, −0.01 per 1 tertile increase in score); and lower risk of abdominal obesity (EWG: OR 0.55, CI 0.43, 0.70 third versus the first tertile), (Mediterranean Diet: OR 0.60, CI 0.46, 0.76 third versus the first tertile), but was not associated with weight changes (EWG: β 0.14, CI −0.07, 0.36 per 1 tertile increase in score), (Mediterranean Diet: β 0.03, CI −0.19, 0.25 per 1 tertile increase in score) or risk of becoming overweight or obese (EWG: OR 1.09, CI 0.77, 1.52 third versus the first tertile), (Mediterranean Diet: OR 0.91, CI 0.65, 1.27 third versus the first tertile).

**Conclusions:**

The results suggest that adherence to either the Mediterranean Diet or the EWG can help to prevent abdominal obesity in post-menopausal women.

## Introduction

Weight gain, particularly abdominal obesity, is prevalent among women in menopause,^1,2^ and 66–69% of women over 45 in the UK are overweight or obese.^3^ Weight gain is considered age- and lifestyle-related; however, the drop in estrogen during menopause influences the fat distribution, particularly in the abdominal area.^1,4^ Abdominal obesity is associated with adverse metabolic events, including cardiovascular disease, the leading cause of death in post-menopausal women.^1^ Poor dietary quality is an important modifiable factor in the prevention of obesity, and improved dietary quality has been associated with a lower risk of overweight or obesity in both men and women.^5,6^ There are few studies on dietary patterns in post-menopausal women, but the limited evidence suggests that improvements in diet quality are associated with smaller increases in weight and waist circumference (WC); however, the optimum dietary pattern is undecided.^7–9^ This study examines how adherence to the Mediterranean Diet and the Eatwell Guide (EWG) influences weight and WC in post-menopausal women in a UK cohort.

## Experimental methods

### Study population

The UK Women’s Cohort Study (UKWCS) was initially established to investigate the relationships between diet and chronic disease, particularly cancer, and this cohort’s complete details have been published.^10^ A total of 7859 post-menopausal participants were identified from their answers on the baseline questionnaire, and 4162 were selected after the following exclusions: 1760 had missing (*n* = 1756) or implausible (*n* = 4) anthropometric data; 375 had implausible daily energy intake of less than 500 kcals or more than 3500 kcals per day,^11^ and a further 1562 had missing confounding variables (*n* = 1536) or discordant waist measurements (*n* = 26).

### Dietary assessment

Dietary information was obtained from a self-administered 217-item validated Food Frequency Questionnaire (FFQ). These values were then used to generate a score for each participant for the EWG and the Mediterranean diet. The Mediterranean Diet score was based on the original score described by Trichopoulou, Kouris-Blazos,^12^ which was adapted for use with the UKWCS dataset.^13^ The median value used to derive the Mediterranean Diet score is shown in Table 1. Adherence to the EWG was assessed the same way as previously used in a study by Scheelbeek, Green.^14^ The dietary intake of each participant was compared to the recommended intake in the EWG except for the values for total fat, which were compared to the Public Health England (PHE) government dietary recommendations.^15,16^

**Table 1.** Median values used for derivation of the Mediterranean Diet score.

	Indicator value	
MDS component	1	0
Vegetables (g/day)	≥294.4	<294.4
Legumes (g/day)	≥29.4	<29.4
Fruit and nuts (g/day)	≥302.7	<302.7
Cereals (g/day)	≥222.0	<222.0
Fish (g/day)	≥26.0	<26.0
MUFA + PUFA: SFA	≥1.53	<1.53
Meat (g/day)	<39.9	≥39.9
Poultry (g/day)	<12.9	≥12.9
Dairy (g/day)	<102.8	≥102.8
Alcohol (g/day)	5–25	<5 or >25

### Anthropometric measurements

Anthropometric measurements were recorded from the baseline and Phase 2 questionnaire and were self-reported measurements on WC, height and weight. Participants were categorised into abdominal obesity categories based on their WC, where abdominal obesity was classified as having a WC of ≥88 cm. Participants were also categorised into weight categories based on their BMI, where a BMI over 25 kg/m^2^ was classified as overweight or obese.

### Covariate measurements

Demographic and socioeconomic information was self-reported in the baseline questionnaire. The variables controlled for were age, physical activity, education, smoking and use of Hormone Replacement Therapy (HRT). These were thought to have links between dietary patterns and obesity and have been controlled for in previous studies.^17,18^ Although ethnicity was identified as a potential confounder, it was not included in this analysis as the majority (99.3%) of the participants selected for this study, who supplied their ethnicity, were white.

### Statistical analysis

All statistical analysis was conducted using the Statistical Package for the Social Sciences (SPSS),^19^ and statistical significance was reported as <0.05.

Hierarchical multiple linear regression models were used to evaluate the association between the Mediterranean Diet and EWG scores (by tertile of adherence and as a continuous scale) and changes in WC (cm; continuous) from baseline to Phase 2. The first model was minimally adjusted for age (years; continuous) and baseline WC (cm; continuous); the second model included adjustments for total energy intake (kcal; continuous), time from baseline to Phase 2 (year; continuous), physical activity (met the physical activity recommendations Yes/No; dichotomous), smoking (never/current/former; nominal), education (No qualifications, O Levels, A levels, Degree; nominal) and HRT (never/current/former; nominal). Finally, the model was adjusted for changes in BMI (kg/m2 continuous) to understand how weight changes explained any differences. Hierarchical linear regression was then repeated to look at the association between adherence to dietary patterns and changes in weight. All the same adjustments were made, except baseline WC was replaced with baseline BMI in the first model.

For those with a normal (<88 cm) WC at baseline, the relationship between dietary scores and risk of abdominal obesity was assessed using binary logistic regression for each one-point increase in score (continuous) and tertile increase in score (categorical). The first model was minimally adjusted for age, and the second model included adjustments for total energy intake (kcal; continuous), time from baseline to Phase 2 (years; continuous), physical activity (Yes/No; nominal), smoking (never/current/former; nominal), education (none/O level/A level/Degree; nominal) and HRT (never/current/former; nominal). The binary logistic regression was then repeated for those with a BMI of less than 25 kg/m^2^ to investigate the relationship between adherence to dietary patterns and the risk of becoming overweight or obese.

## Results

After a mean of 4.1 (SD 0.7) years, the mean weight increase across all participants was 1.2 (SD 4.8) kg, and the mean increase in WC was 6.7 (SD 6.8) cm. At baseline, the prevalence of abdominal obesity was 7.7%, and at Phase 2, 24.4%. The prevalence of overweight or obese participants was 32.3% at baseline, and at Phase 2, 37.5%. Weight, WC, BMI, time from baseline to Phase 2 and the percentage of participants with general and abdominal obesity decreased along the tertiles for the Mediterranean Diet and the EWG. The percentage meeting the requirements for physical activity and having higher qualifications also increased along the tertiles. In addition, those in the highest tertile of adherence for the Mediterranean diet were younger and less likely to smoke, and those in the highest tertile for the EWG had a smaller increase in WC (Tables 2 and 3).

**Table 2.** Characteristics of participants according to tertiles of adherence to the Mediterranean Diet. Continuous variables are presented as the median and interquartile range (IQR) and categorical variables as percentages p-values obtained from the Kruskal–Wallis H test for continuous variables and Chi-squared test for categorical variables.

	Mediterranean Diet score tertiles (0–10)						
	1^st^ (*n* = 1008)		2^nd^ (*n* = 2223)		3^rd^ (*n* = 931)		
	(0–3)		(4–6)		(7–10)		
Variables	Median	IQR	Median	IQR	Median	IQR	*p*
Age (years)	58.1	10.1	57.6	10.0	57.2	10.4	0.03
Time from baseline (years)	4.0	0.4	3.9	0.5	3.9	0.7	0.002
Weight baseline (kg)	64.4	11.4	64.0	12.7	62.0	12.2	<0.001
Weight Phase 2 (kg)	65.3	14.1	64.0	12.7	63.5	12.8	<0.001
Weight change (kg)	0.9	4.5	0.9	4.1	0.9	4.1	0.40
WC baseline (cm)	76.1	12.7	73.7	10.2	71.1	7.6	<0.001
WC Phase 2 (cm)	81.5	14.6	80.0	13.3	78.7	14.0	<0.001
WC difference (cm)	5.7	8.3	5.7	7.6	5.7	7.6	0.31
BMI baseline (kg/m^2^)	24.0	4.5	23.4	4.2	23.0	4.0	<0.001
BMI Phase 2 (kg/m^2^)	24.5	4.6	23.9	4.5	23.4	4.5	<0.001
BMI change (kg/m^2^)	0.4	1.7	0.3	1.6	0.3	1.5	0.42
	%		%		%		
Physical activity^a^	42.9		52.3		59.8		<0.001
HRT							
Never	52.5		54.3		56.0		0.44
Current		33.2		32.1		29.5	
Past	14.3		13.6		14.5		
Education							
No formal	19.3		17.9		16.2		<0.001
O level	35.7		30.5		24.9		
A level	25.3		27.1		29.3		
Degree or above	19.6		24.5		29.5		
Smoking							
Never	65.5		61.8		51.5		<0.001
Current	8.8		6.9		5.5		
Former	25.7		31.3		39.4		
Abdominal obesity baseline^b^	10.8		7.2		5.5		<0.001
Abdominal obesity Phase 2^b^	30.4		23.4		20.2		<0.001
Overweight or obese baseline^c^	38.3		32.4		25.7		<0.001
Overweight or obese Phase 2^c^	43.0		36.8		33.2		<0.001

IQR: interquartile range; WC: waist circumference; BMI: body mass index; HRT: hormone replacement therapy.

^a^Physical activity, percentage meeting recommendations.

^b^Abdominal obesity WC >88 cm.

^c^Overweight or obese ≥25 kg/m^2^.

**Table 3.** Characteristics of participants according to tertials of adherence to the Eatwell Guide. Continuous variables are presented as the median and interquartile range, categorical variables as percentages-p values obtained from the Kruskal–Wallis H test for continuous variables and the Chi-squared test for categorical variables.

	EWG tertiles (0–9)						
	1^st^ (*n* = 1205)		2^nd^ (*n* = 1988)		3^rd^ (*n* = 969)		
	(0–2)		(3–4)		(5–9)		
Variables	Median	IQR	Median	IQR	Median	IQR	*p*
Age (years)	57.8	10.1	57.5	10.2	57.7	10.0	0.79
Time from baseline (years)	4.0	0.4	3.9	0.5	3.9	0.7	0.02
Weight baseline (kg)	63.5	12.2	63.5	12.7	62.1	13.6	<0.001
Weight Phase 2 (kg)	65.3	14.5	63.5	12.7	63.5	13.6	<0.001
Weight change (kg)	0.9	4.0	0.9	4.1	0.9	4.5	0.20
WC baseline (cm)	76.2	10.2	71.1	10.2	71.1	7.6	<0.001
WC Phase 2 (cm)	81.5	14.4	80.0	13.8	78.7	13.6	<0.001
WC difference (cm)	6.3	8.3	5.7	7.6	5.1	7.6	0.01
BMI baseline (kg/m^2^)	23.9	4.6	23.4	4.0	23.2	4.0	<0.001
BMI Phase 2 (kg/m^2^)	24.1	4.6	24.0	4.7	23.7	4.4	0.001
BMI change (kg/m^2^)	0.3	1.5	0.3	1.6	0.4	1.6	0.20
	%		%		%		
Physical activity^a^	46.3		53.4		54.9		<0.001
HRT							
Never	54.1		55.1		52.7		0.62
Current	32.7		30.9		32.4		
Past	13.2		14.0		14.9		
Education							
No formal	17.2		17.3		19.9		0.40
O level	31.5		31.1		28.1		
A level	27.6		26.8		27.2		
Degree or above	23.7		24.8		24.8		
Smoking							
Never	61.5		61.5		60.3		0.05
Current	8.0		7.4		5.3		
Former	30.5		31.1		34.5		
Abdominal obesity baseline^b^	10.3		6.8		6.3		<0.001
Abdominal obesity Phase 2^b^	30.1		23.5		18.9		<0.001
Overweight or obese baseline^c^	37.2		31.3		28.4		<0.001
Overweight or obese Phase 2^c^	40.3		37.2		34.6		0.02

EWG: Eatwell Guide; IQR: interquartile range; WC: waist circumference; BMI: body mass index; HRT: hormone replacement therapy.

^a^Physical activity, percentage meeting recommendations.

^b^Abdominal obesity WC >88 cm.

^c^Overweight or obese ≥25 kg/m^2^.

Linear regression analysis identified a significant negative association between an increase in Mediterranean Diet and EWG score and changes in WC in all fully adjusted models (Table 4). The association between the EWG score and WC was stronger than that seen with the Mediterranean Diet. No significant associations were seen between adherence to the Mediterranean Diet or the EWG and changes in weight (Table 5).

**Table 4.** Multiple linear regression models describing the association between an increase in Mediterranean Diet Score or EWG score (continuous variable, per tertile increase) and change in waist circumference between baseline and Phase 2 (β coefficients and 95% confidence intervals).

	β	95% CI	*p*
Mediterranean Diet (tertiles)			
Model 1^a^	−0.27	−0.57, 0.04	0.08
Model 2^b^	−0.25	−0.57, 0.06	0.12
Model 3^c^	−0.29	−0.58, −0.01	0.05
Mediterranean (continuous)			
Model 1^a^	−0.11	−0.22, −0.004	0.04
Model 2^b^	−0.11	−0.22, 0.01	0.07
Model 3^c^	−0.12	−0.23, −0.02	0.02
EWG (tertiles)			
Model 1^a^	−0.38	−0.67, −0.09	0.01
Model 2^b^	−0.38	−0.68, −0.08	0.01
Model 3^c^	−0.47	−0.75, −0.20	0.001
EWG (continuous)			
Model 1^a^	−0.19	−0.33, −0.06	0.01
Model 2^b^	−0.20	−0.35, −0.05	0.01
Model 3^c^	−0.24	−0.38, −0.12	<0.001

CI: confidence intervals; EWG: Eatwell Guide.

^a^Model 1 includes age and baseline waist circumference.

^b^Model 2 additionally includes the time from baseline to Phase 2, total energy intake, smoking, education, physical activity, smoking and HRT.

^c^Model 3 additionally includes BMI changes from baseline to Phase 2.

**Table 5.** Multiple linear regression models describing the association between an increase in Mediterranean Diet Score or Eatwell Guide score (continuous variable, per tertile increase) and change in weight between baseline and Phase 2 (β coefficients and 95% confidence intervals).

	β	95% CI	*p*
Mediterranean Diet (tertiles)			
Model 1^a^	−0.05	−0.27, 0.16	0.64
Model 2^b^	0.03	−0.19, 0.25	0.80
Mediterranean Diet (continuous)			
Model 1^a^	−0.14	−0.09, 0.06	0.71
Model 2^b^	0.16	−0.06, 0.10	0.70
EWG (tertiles)			
Model 1^a^	0.20	−0.001, 0.41	0.05
Model 2^b^	0.14	−0.07, 0.36	0.20
EWG (continuous)			
Model 1^a^	0.09	−0.004, 0.19	0.06
Model 2^b^	0.06	−0.04, 0.17	0.23

CI: confidence intervals; EWG: Eatwell Guide.

^a^Model 1 includes age and baseline BMI.

^b^Model 2 additionally includes the time from baseline to Phase 2, total energy intake, smoking, education, physical activity, smoking and HRT.

Binomial regression models identified that a higher index score for both the Mediterranean Diet and the EWG was associated with a reduced risk of becoming abdominally obese in all models (Table 6). However, a higher index score was not significantly associated with the risk of becoming overweight or obese (Table 7).

**Table 6.** Binomial logistic regression models describing the relationship between adherence to the Mediterranean Diet and Eatwell Guide and becoming abdominally obese in participants with a waist circumference of less than 88 cm at baseline. (Odds ratio and 95% confidence intervals).

	OR^a^	95% CI	OR^b^	95% CI
Mediterranean Diet				
Tertile 1 (0–3)	1	Reference	1	Reference
Tertile 2 (4–6)	0.76	0.63, 0,92	0.74	0.61, 0.90
Tertile 3 (7–10)	0.65	0.52, 0.83	0.60	0.46, 0.76
Continuous (0–10)	0.91	0.88, 0.95	0.90	0.86, 0.94
EWG				
Tertile 1 (0–2)	1	Reference	1	Reference
Tertile 2 (3–4)	0.74	0.62, 0.89	0.75	0.62, 0.90
Tertile 3 (5–9)	0.54	0.42, 0.68	0.55	0.43, 0.70
Continuous (0–9)	0.84	0.80, 0.90	0.84	0.80, 0.90

OR: odds ratio; CI: confidence intervals; EWG: Eatwell Guide.

^a^Adjusted for age.

^b^Further adjusted for the time from baseline to Phase 2, total energy intake, smoking, education, physical activity and HRT.

**Table 7.** Binomial logistic regression models describing the relationship between adherence to the Mediterranean Diet and Eatwell Guide and becoming overweight or obese in participants with a weight less than 25 kg/m^2^ at baseline. (Odds ratio and 95% confidence intervals).

	OR^a^	95% CI	OR^b^	95% CI
Mediterranean Diet				
Tertile 1 (0–3)	1	Reference	1	Reference
Tertile 2 (4–6)	0.82	0.63, 1.08	0.86	0.65, 1.13
Tertile 3 (7–10)	0.86	0.63, 1.18	0.91	0.65, 1.27
Continuous (0–10)	0.97	0.92, 1.03	0.98	0.92, 1.04
EWG				
Tertile 1 (0–2)	1	Reference	1	Reference
Tertile 2 (3–4)	1.20	0.92, 1.56	1.19	0.91, 1.57
Tertile 3 (5–9)	1.17	0.85, 1.60	1.09	0.77, 1.52
Continuous (0–9)	1.03	0.95, 1.11	1.01	0.93, 1.10

OR: odds ratio; CI: confidence intervals; EWG: Eatwell Guide.

^a^Adjusted for age.

^b^Further adjusted for the time from baseline to Phase 2, total energy intake, smoking, education, physical and HRT.

## Discussion

This study has found that higher adherence to the EWG and the Mediterranean Diet is associated with lower gains in WC and a reduced risk of abdominal obesity in post-menopausal women. Cespedes Feliciano, Tinker^7^ found similar results in their prospective cohort study of post-menopausal women. They examined four different dietary indices, including those based on the American Healthy Eating Guidelines adapted to incorporate more foods predictive of preventing disease (AHEI-2010) and the Alternate Mediterranean Diet Score (AMDS). They found that each 10% increase in dietary quality score was associated with between 0.10 cm (AMDS) and 0.20 cm (AHEI-2010) smaller increases in WC. A prospective cohort study of 32,119 men and women in Italy also observed that increased adherence to the Italian Mediterranean Diet was significantly associated with negative changes in WC and a reduced risk of becoming abdominally obese.^18^

Similarly, in Spain, increased adherence to the Mediterranean Diet was associated with smaller WC increases after 10 years. In addition, they also saw a decreased incidence of abdominal obesity, but this did not reach significance.^20^ Cross-sectional studies have also observed an association with adherence to the Mediterranean Diet and lower WCs^17,21^ and a reduced risk of abdominal obesity with higher adherence to the Healthy Eating Index in America^22^; however, in a study of Mexican Americans, the improvements in diet quality were associated with a lower risk of abdominal obesity in men but not in women.^23^

No significant associations were seen between adherence to the Mediterranean Diet or the EWG and weight changes or the risk of becoming overweight or obese in those with a BMI of less than 25 kg/m^2^ at baseline. Similar results for weight gain have been seen previously in post-menopausal women where adherence to the Mediterranean Diet was not significantly associated with weight gain in fully adjusted models, and adherence to AHEI-2010 was associated with a higher risk of gaining weight.^8^ An increase in adherence to the Mediterranean Diet was also not significantly associated with changes in weight over 5 years in an extensive study of both men and women in Italy. However, when the results were stratified by BMI, a significant weight reduction was seen in those with a BMI less than 25 kg/m^2^ ^18^. Cross-sectional studies have also not found a significant association between healthy eating patterns and BMI.^17,21^

In contrast to this study, some other studies have shown that adherence to the Mediterranean Diet is associated with reduced weight gain ^24^ and a reduced likelihood of becoming overweight or obese.^18,24^ However, in the multicentre, prospective study, significant heterogeneity was seen between countries, and one study in the UK saw a non-significant increase in weight gain.^24^ These conflicting results are a possible indication that there may be variations in the diet in the UK compared to Mediterranean regions, and a similar lack of association between adherence to the Mediterranean Diet and weight was seen in a younger population in Sweden.^25^ Differences in results with the Mediterranean Diet may also be linked to differences in the scores. This study’s score was based on median values specific to the population, so results are not directly comparable between studies.^26^

The strength of this study is the availability of baseline and follow-up data from a large prospective cohort, the use of validated questionnaires for the dietary intake alongside the collection of additional data on potential confounders used in the regression models. This study, however, does have several limitations. The anthropometric measurements were self-reported, and the FFQ was administered only on a single occasion at baseline. In addition, the cohort’s population is generally healthier,^27^ and the study is limited to those who returned the Phase 2 questionnaire and those who had complete and plausible data.

The results of this study add to the paucity of evidence in this area and suggest that adhering to dietary guidelines can help prevent abdominal adiposity in post-menopausal women. Adherence to guidelines in the UK is currently very low. For higher adherence, women need to consume more fibre, fruit, vegetables and oily fish and less free sugars and saturated fats.^28^ Current recommendations are that public health interventions should routinely include diet and lifestyle advice alongside appropriate HRT prescribing at perimenopause. Doing this could limit the adverse health implications seen in post-menopausal women and reduce the levels of avoidable health issues in the female population.^29,30^
